# Patellar Denervation with Electrocautery Reduces Anterior Knee Pain within 1 Year after Total Knee Arthroplasty: A Meta‐Analysis of Randomized Controlled Trials

**DOI:** 10.1111/os.12735

**Published:** 2020-12-22

**Authors:** Ming‐cheng Yuan, Zi‐chuan Ding, Ting‐xian Ling, Zongke Zhou

**Affiliations:** ^1^ Department of Orthopedics West China Hospital/West China School of Medicine, Sichuan University Chengdu 610041 China

**Keywords:** Anterior knee pain, Meta‐analysis, Patellar denervation with electrocautery, Total knee arthroplasty

## Abstract

**Objective:**

The effect of patellar denervation with electrocautery (PD) on anterior knee pain (AKP) after total knee arthroplasty (TKA) is still debated. The aim of this meta‐analysis was to evaluate the current evidence regarding the use of PD in TKA without patellar resurfacing.

**Methods:**

A computerized search of published studies was performed in the PubMed, Embase and Cochrane Library databases in December 2019. Eligible studies were randomized controlled trials (RCTs) comparing clinical outcomes of the PD group and the non‐PD group. Subgroup analyses were carried out according to the follow‐up time (3, 12 months, and over 12 months) to evaluate whether the clinical effect of PD changed with time.

**Results:**

Ten RCTs were included in this meta‐analysis. Pooled results showed a lower rate of AKP (Risk Ratio [RR] = 0.70; 95% confidence interval [CI], 0.50 to 0.97; *P* = 0.03) and a reduction in visual analogue scale (VAS) for AKP (mean difference, −0.37; 95% CI, −0.69 to −0.05; *P* = 0.02) in the PD group when compared to the non‐PD group. Subgroup analyses found the differences in AKP incidence and VAS for AKP were significant at 3‐ and 12‐month follow‐up but not after 12‐month follow‐up. No significant difference was observed in functional scores between the two groups. No specific complication directly or indirectly related to PD was found.

**Conclusion:**

PD can decrease the incidence and severity of AKP within 12 months after TKA, but the effect cannot be maintained after 12‐month follow‐up. Without significant associated complication and reoperation, the use of PD is still recommended in TKA without patellar resurfacing.

## Introduction

Total knee arthroplasty (TKA) is considered to be the successful treatment of choice for end‐stage knee osteoarthritis, and can provide excellent postoperative pain relief, remarkable deformity correction, and satisfactory function recovery. However, residual anterior knee pain (AKP) after TKA has been a common and persistent complaint, and results in dissatisfaction and low quality of life of patients^1^, ^2^. The reported incidence of AKP ranges from 17.5% to 29.0%^3^, ^4^, ^5^. The underlying cause for AKP still remains unclear, but its development was believed to be associated with the presence of substance P nociceptive afferent fibers in the peripatellar soft tissues^6^, ^7^.

Theoretically, patellar denervation with electrocautery (PD) can disable the pain receptors, interrupt the pain pathways, achieve denervation of the anterior knee region, and consequently prevent AKP after TKA. PD has been widely performed among surgeons who do not carry out patellar resurfacing in TKA. It has been reported that 56% of the surgeons in the Netherlands routinely use PD to prevent AKP in TKA without patellar resurfacing^8^. However, the exact effect of PD on the incidence and severity of AKP after TKA is still debated. A number of randomized controlled trials (RCTs) regarding the effect of PD have been published with controversial results. Some indicated that PD has no clear advantage over non‐patellar denervation (non‐PD)^9^, ^10^, while some believed PD can reduce incidence of AKP and improve knee function after TKA^11^, ^12^. Moreover, several recent studies found that PD can improve clinical outcomes at the early stage of postoperative period but the effect cannot be maintained after a longer follow‐up time, suggesting that the clinical effect of PD on TKA changed in a time‐dependent manner^13^, ^14^, ^15^.

The literature search in the most recently published meta‐analysis on this topic by Zhang *et al* ^16^. was conducted in February 2015. A total of six RCTs were included in the previous meta‐analysis and the authors concluded that PD has a superior effect on clinical outcomes than non‐PD. Another meta‐analysis including seven RCTs by Xie *et al*. found PD can significantly reduce AKP and improve early knee function after TKA^17^. But the advantages disappear after a prolonged period of follow‐up. Importantly, several additional RCTs have been published in this field in recent years. In order to draw an updated conclusion to help orthopaedic surgeons make a wiser clinical decision, a meta‐analysis of RCTs was performed to investigate the effect of PD on AKP after TKA without patellar resurfacing, and to determine whether the clinical effect of PD changes with time.

## Materials and Methods

The meta‐analysis performed adhered to the Preferred Reporting Items for Systematic Reviews and Meta‐Analyses (PRISMA) guidelines^18^ and AMSTAR (Assessing the Methodological Quality of Systematic Reviews) Guidelines^19^.

### *Search Strategy*

A computerized search was performed in the PubMed, Embase and Cochrane Library databases in December 2019 for studies published. No time frame was specified with respect to publication date and there was no language restriction. The following keywords were used along with the Boolean operator: Denervation, Electrocautery, Knee arthroplasty. Details of the search strategy are shown in Table 1. Manual search of bibliographies from reviews and selected studies was also performed for additional studies.

**TABLE 1:** Details of search strategy

Database	Search strategy
Pubmed	(Denervation[tiab] OR Electrocautery[tiab] OR Electrocoagulation[tiab] OR Denervation[MeSH] OR Electrocoagulation[MeSH]) AND (Knee arthroplasty[tiab] OR Knee replacement[tiab] OR Arthroplasty, Replacement, Knee[MeSH])
Embase	#1 “Denervation”:ab,ti OR “Electrocautery”:ab,ti OR “Electrocoagulation”:ab,ti #2 “Denervation”/exp. #3 “Electrocoagulation”/exp. #4 “Knee arthroplasty”:ab,ti OR “Knee replacement”:ab,ti #5 “Knee arthroplasty”/exp. #6 (#1 OR #2 OR #3) #7 (#4 OR #5) #8 (#6 AND #7)
Cochrane Library	#1 Denervation OR Electrocautery OR Electrocoagulation:ti,ab,kw #2 MeSH descriptor: [Denervation] explode all trees #3 MeSH descriptor: [Electrocoagulation] explode all trees #4 Knee arthroplasty OR Knee replacement:ti,ab,kw #5 MeSH descriptor: [Arthroplasty, Replacement, Knee] explode all trees #6 (#1 OR #2 OR #3) #7 (#4 OR #5) #8 (#6 AND #7)

### *Article Selection*

The inclusion criteria were: (i) patients underwent primary TKA without patellar resurfacing; (ii) PD were performed in the experimental group; (iii) non‐PD were performed in the control group; (iv) follow‐up data about postoperative AKP or knee function were provided; and (v) randomized controlled trials. Exclusion criteria were: (i) studies which included patients with knee deformity, lower limb fracture, or previous surgery of lower limb.

Two reviewers independently assessed the titles and abstracts for initial screening of studies, and then the full texts of articles selected from initial screening were evaluated. Disagreement was resolved by discussion and consensus. When the decision was still not reached, a third reviewer's opinion was sought.

### *Assessment of Risk of Bias*

The risk of bias of RCT was graded as high, low, or unclear according to the Cochrane Risk of Bias Tool based on the following domains: selection bias, performance bias, detection bias, attrition bias, reporting bias, and other bias^20^. Other bias was defined as the study with unbalanced baseline characteristics of patients between the PD and non‐PD group. Two reviewers independently assessed the risk of bias of included studies. Any disagreement between reviewers was resolved by a third reviewer.

### *Study Outcomes*

#### *Incidence of Anterior Knee Pain (AKP)*

The primary outcome measure of interest was the incidence of AKP after TKA, which was the proportion of patients having AKP after TKA. AKP has been the most common complaint after TKA, and often results in dissatisfaction and low quality of life of patients^1^, ^2^. The reported incidence of AKP ranges from 17.5% to 29.0%^3^, ^4^, ^5^.

#### *Visual Analogue Scale (VAS) for Anterior Knee Pain (AKP)*

The visual analogue scale (VAS) was the secondary outcome of this study. The visual analogue scale (VAS) is the most commonly used method for measuring pain. For measurement of the magnitude of pain, the most used scale is “no pain” (corresponding to the scale of 0) and “pain too intense to be tolerated” (corresponding to the scale of 10). The severity of the AKP after TKA is evaluated by VAS in most studies regarding the use of PD in TKA.

#### *Range of Motion (ROM)*

The range of motion (ROM) was the secondary outcome of this study. ROM is the degree of flexion and extension of the knee, which is of vital importance to reflect the knee function after TKA.

#### *Patellar Score (PS)*

The Patellar Score (PS) was the secondary outcome of this study. The PS is a questionnaire which includes items on anterior knee pain, quadriceps strength, and ability to rise from a chair and climb stairs; these scores range from 3 to 30 points, with 30 points representing the best score.

#### *American Knee Society Score (KSS)*

The secondary outcomes included American Knee Society Score (KSS), which consists of knee score and function score, each with a maximum of 100 points. The American Knee Society knee score (KSS knee) evaluates the knee joint based on pain, stability and range of motion, with deduction for flexion contracture, extension lag and malalignment. The American Knee Society function score (KSS function) scores the patient's ability to walk and climb stairs, with deduction for aids.

#### *Oxford Knee Score (OKS)*

The secondary outcomes included Oxford knee score (OKS), which is a patient‐reported outcome measure that consists of 12 questions about an individual's activities of daily living and how they have been affected by pain over the last 4 weeks. It is a reliable tool to evaluate the pain and function of the knee after TKA.

#### *Postoperative Complication and Reoperation*

All postoperative complications and reoperations reported in the included studies were extracted.

### *Data Extraction*

Lead author, publication year, country of origin, participant characteristics (age and gender), depth of electrocautery, surgical approach, follow‐up time, and study conclusion were extracted from each included study by two independent reviewers.

### *Statistical Analysis*

Risk Ratio (RR) and 95% confidence intervals (CI) were calculated for dichotomous outcomes using the Mantel–Haenszel (M‐H) method. Mean difference (MD) and 95% CI were calculated for continuous outcomes using the Inverse Variance (IV) method. If the original study did not report standard deviation (SD), we estimated SD according to Hozo *et al* ^21^. A random effects model was used to pool the data. Heterogeneity between studies was measured by the I^2^ statistics (I^2^ > 50% representing significant heterogeneity). If zero event was reported for one group in a comparison, a value of 0.5 was added to both groups for each such study. If studies reported zero events in both groups, the data was not included in the meta‐analysis^22^. To evaluate whether the clinical effect of PD changes with time, subgroup analyses of the function outcomes were carried out according to follow‐up time (3 months, 12 months, and over 12 months; Table 3). Outcomes with only one study reporting were not included in subgroup analyses. Besides, subgroup analysis was carried out according to different depths of electrocautery. Publication bias was assessed by generating funnel plots. The level of significance was defined as *P* < 0.05. Statistical analyses were performed by using Review Manager 5.3.

## Results

### *Article Selection and Characteristics*

The initial literature search identified 1198 studies, and manual search yielded one additional study^23^. After duplicates were removed, titles and abstracts of 664 studies were assessed. The full texts of 42 studies were then evaluated, and ultimately 10 studies that met the inclusion criteria were included in this meta‐analysis^9^, ^10^, ^11^, ^12^, ^13^, ^14^, ^15^, ^23^, ^24^, ^25^.

**Fig. 1:**
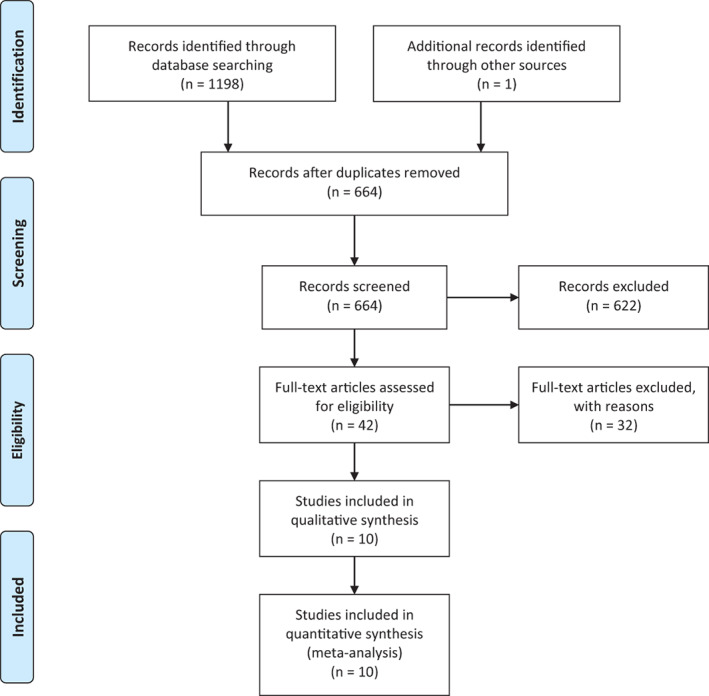
Flow diagram of included studies.

The flow diagram is presented in Fig. 1. Two studies by van Jonbergen *et al*. from the same cohort were included as they had different follow‐up times (12 and 44 months)^14^, ^15^. The earlier study in 2011 with 12‐month follow‐up was included in the subgroup analysis^14^.

Characteristics of included studies are summarized in Table 2. The 10 included RCTs were published from 2004 to 2019, with sample sizes ranging from 38 to 262 subjects. Participants had unilateral TKA procedures in all studies except one by Yim *et al*. (bilateral TKA)^24^. The age and gender of participants were comparable between PD and non‐PD group in all studies that reported related details. The depths of electrocautery in PD group was varied among studies: from within 1 mm to within 10 mm. Eight of the 10 included studies used medial parapatellar approach during TKA procedure and two of them used midvastus approach. The follow‐up time of included studies ranged from 10 to 64 months.

**TABLE 2:** Details of included studies

References	Country	No. of knees			Age		Gender (female)		Depth of electrocautery	Approach	Follow‐up time (mean; months)	Conclusion
		PD	NPD	Total	PD	NPD	PD	NPD				
Alomran, 2015^12^	Saudi Arabia	92	92	184	NA	NA	NA	NA	Within 2 mm	MP	37.4 (PD); 39.0 (NPD)	38‐Month result: favor PD
Altay *et al*., 2012^11^	Turkey	35	35	70	68	68	74.3%	74.3%	2–3 mm	MV	36 (24–60)	36‐Month result: favor PD
Baliga, 2012^9^	United Kingdom	91	94	185	69.0	69.2	44.0%	55.3%	Within 1 mm	MP	12	3‐, 6‐ and 12‐Month results: neutral
Kwon *et al*., 2015^10^	Korea	50	50	100	66.3	67.0	100%	100%	About 1 mm	MP	64.8 (60–70)	3‐, 6‐ and 60‐Month results: neutral
Motififard *et al*., 2018^25^	New Zealand	46	46	92	64.3	68.0	71.7%	64.1%	1–2 mm	MV	10	3‐Week result: favor PD; 3‐, 6‐ and 10‐month results: neutral
Pulavarti *et al*., 2014^13^	United Kingdom	63	63	126	69.9	69.8	50.8%	57.1%	NA	MP	26.5 (PD); 26.3 (NPD)	3‐Month result: favor PD; 12‐ and 24‐month results: neutral
Saoud, 2004^23^	Egypt	19	19	38	NA	NA	NA	NA	1 mm	MP	11 (PD); 10 (NPD)	10‐Month result: favor PD
van Jonbergen *et al*., 2011^14^	The Netherlands	131	131	262	71	72	72.5%	64.1%	Within 1 mm	MP	12	12‐Month result: favor PD
van Jonbergen *et al*., 2014^15^	The Netherlands	103	99	202	70	71	72.8%	65.7%	Within 1 mm	MP	44.4 (12–50)	44‐Month result: neutral
Yim *et al*., 2012^24^	Korea	50	50	100	70.2	70.2	100%	100%	Within 2–3 mm	MP	21 (12–48)	21‐Month result: neutral

MP, medial parapatellar approach; MV, midvastus approach; NA, not available; NPD, non‐patellar denervation; PD, patellar denervation.

**Fig. 2:**
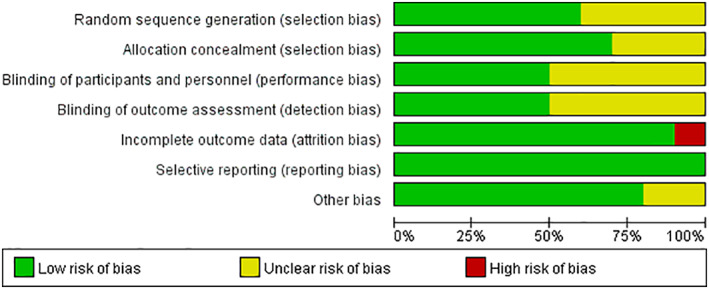
Summary of the risk of bias across the included studies.

**Fig. 3:**
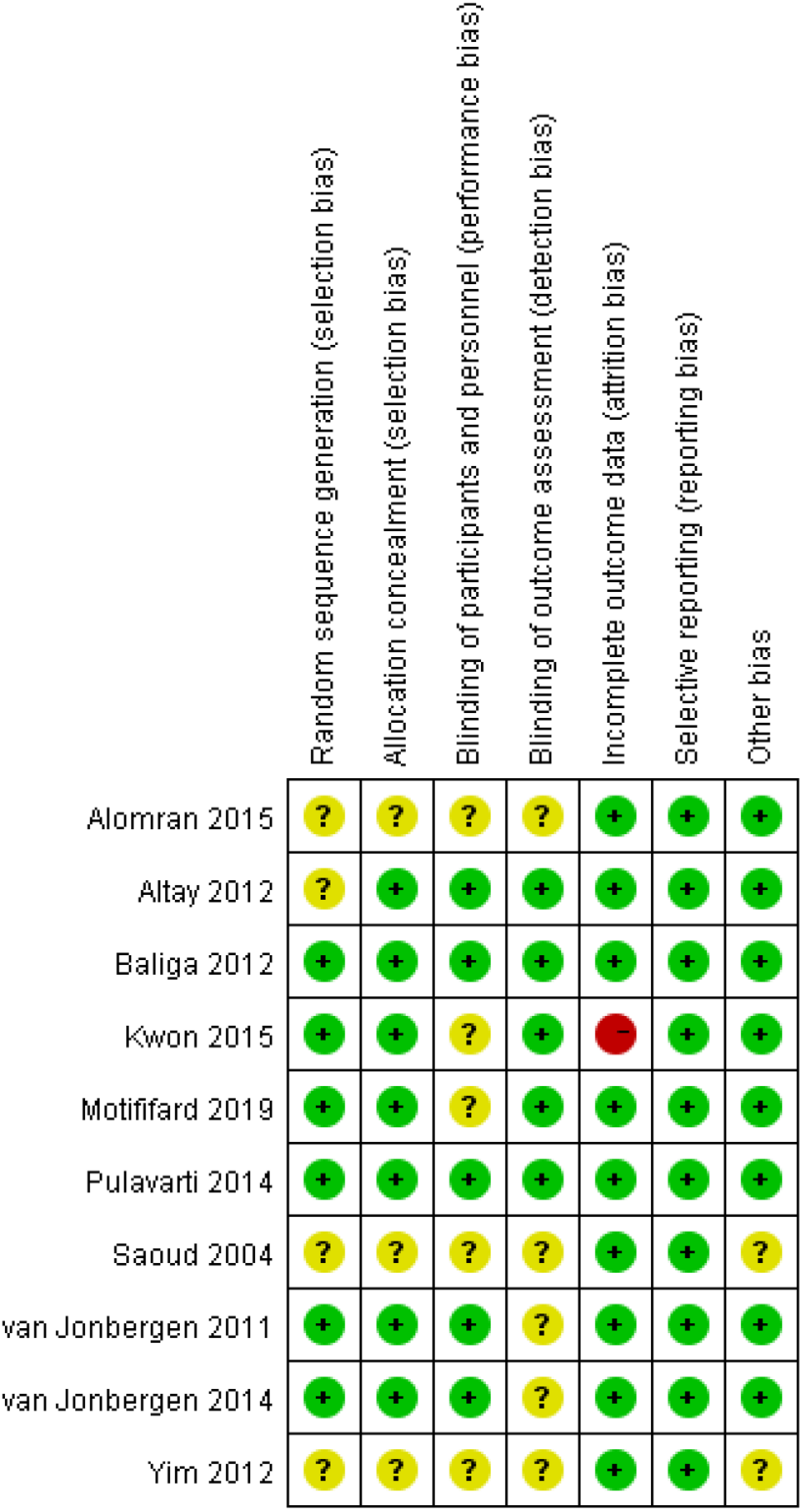
Risk of bias in each study. “+” indicates a low risk of bias; “‐” indicates a high risk of bias; “?” indicates unclear risk of bias.

Details of risk of bias are presented in Figs 2 and 3. Three studies did not provide methodology information about randomization and blinding, and thus were assessed as unclear risk of bias in selection bias, performance bias, and detection bias^12^, ^23^, ^24^. Two studies that did not provide baseline data for both groups were assessed as unclear risk of bias in other bias^23^, ^24^.

### *Meta‐analysis Results*

#### *Incidence of Anterior Knee Pain (AKP）*

**Fig. 4:**
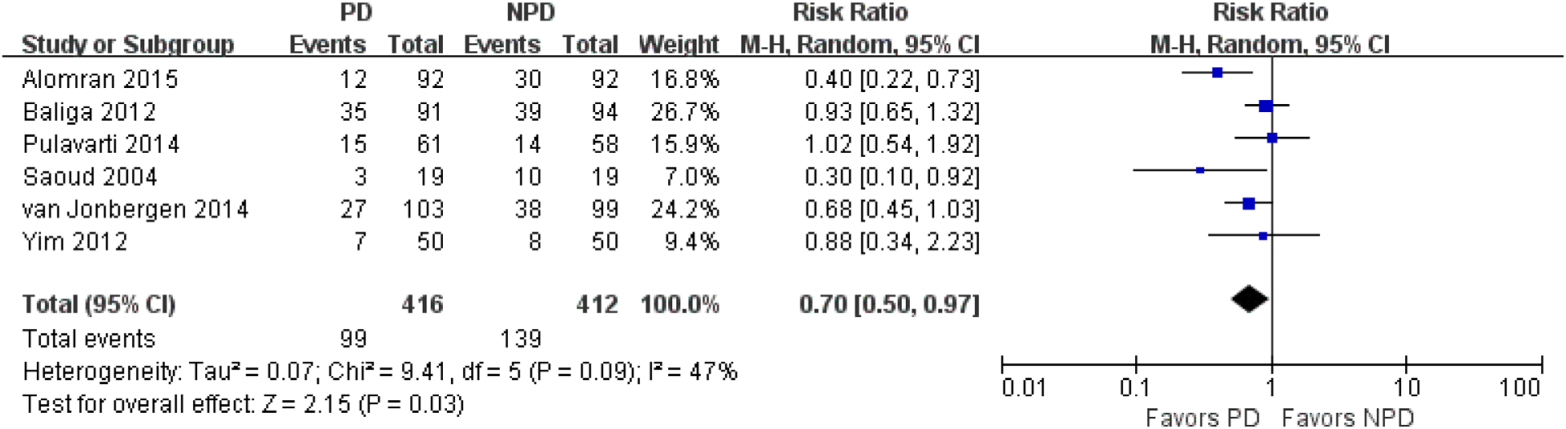
Forest plot of incidence of anterior knee pain (AKP).

Six studies compared the incidence of AKP between PD and non‐PD patients. A total of 99 patients out of 416 patients (23.8%) in the PD group and 139 out of 412 patients (33.7%) in the non‐PD group had AKP. As shown in Fig. 4, PD was associated with significantly lower rate of AKP (RR = 0.70; 95% CI, 0.50 to 0.97; *P* = 0.03), without significant heterogeneity (I^2^ = 47%). In subgroup analysis, PD was associated with significantly lower rate of AKP at 12‐month follow‐up (RR = 0.70; 95% CI, 0.50 to 0.98; *P* = 0.03). No significant difference was observed in AKP incidence between the PD group and non‐PD group after 12‐month follow‐up (RR = 0.68; 95% CI, 0.46 to 1.00; *P* = 0.05) (Table 3).

**TABLE 3:** Results of subgroup analyses of different follow‐up times

Follow‐up time and outcomes	Studies	Participants (No. of knees)			Overall effect	
		PD	NPD	Overall	OR/WMD (95% CI)	*P*
3 Months						
VAS	2	109	109	218	−0.52 (−0.89, −0.14)	0.007
PS	3	159	159	318	1.50 (−0.76, 3.75)	0.19
KSS knee	3	159	159	318	−1.01 (−3.77, 1.75)	0.47
KSS function	2	109	109	218	4.67 (2.07, 7.26)	<0.001
OKS	2	154	157	311	0.66 (−1.19, 2.51)	0.48
12 Months						
AKP	4	304	306	610	0.70 (0.50, 0.98)	0.03
VAS	3	200	202	402	−0.51 (−0.84, −0.19)	0.002
PS	3	159	158	317	0.07 (−0.76, 0.90)	0.88
KSS knee	4	290	289	579	0.21 (−2.06, 2.48)	0.85
KSS function	3	240	239	479	0.81 (−1.75, 3.37)	0.54
OKS	2	154	156	310	−0.18 (−2.92, 2.57)	0.90
Over 12 months						
AKP	4	306	299	605	0.68 (0.46, 1.00)	0.05
VAS	2	96	93	189	−0.31 (−0.82, 0.19)	0.22
ROM	3	188	185	373	7.68 (0.33, 15.04)	0.04
PS	3	146	143	289	0.75 (−0.25, 1.75)	0.14
KSS knee	3	188	184	372	1.83 (−0.28, 3.94)	0.09
KSS function	2	138	134	272	3.66 (0.29, 7.04)	0.03

KSS function, American Knee Society function score; KSS knee, American Knee Society knee score; NPD, non‐patellar denervation; OKS, Oxford knee score; PD, patellar denervation; PS, Patellar score; ROM, range of motion; VAS, visual analogue scale.

#### *Visual Analogue Scale (VAS) for Anterior Knee Pain (AKP)*

**Fig. 5:**
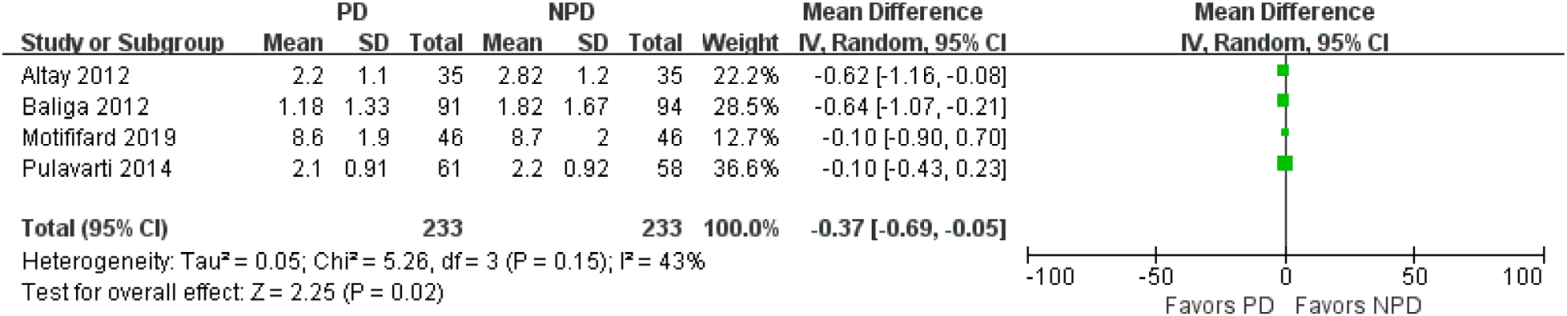
Forest plot of visual analogue scale (VAS) for anterior knee pain.

Four studies involving 466 patients reported the VAS for AKP. PD was associated with a reduction in VAS (mean difference, −0.37; 95% CI, −0.69 to −0.05; *P* = 0.02; Fig. 5). No significant heterogeneity was observed across studies (I^2^ = 43%). In the subgroup analysis, PD was associated with lower VAS at 3‐month follow‐up (mean difference, −0.52; 95% CI, −0.89 to −0.14; *P* = 0.007;) and 12‐month follow‐up (mean difference, −0.51; 95% CI, −0.84 to −0.19; *P* = 0.002). After 12‐month follow‐up, no significant difference was observed in VAS between the PD group and non‐PD group (mean difference, −0.31; 95% CI, −0.82 to 0.19; *P* = 0.22) (Table 3).

#### *Range of Motion (ROM)*

**Fig. 6:**
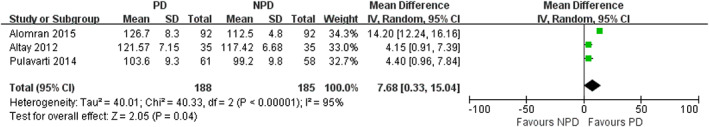
Forest plot of knee range of motion (ROM).

Three studies investigated ROM of the involved knee. PD was associated with significantly higher ROM after 12‐month follow‐up (Mean difference, 7.68; 95% CI, 0.33 to 15.04; *P* = 0.04; Fig. 6; Table 3).

#### *Patellar Score (PS)*

**Fig. 7:**
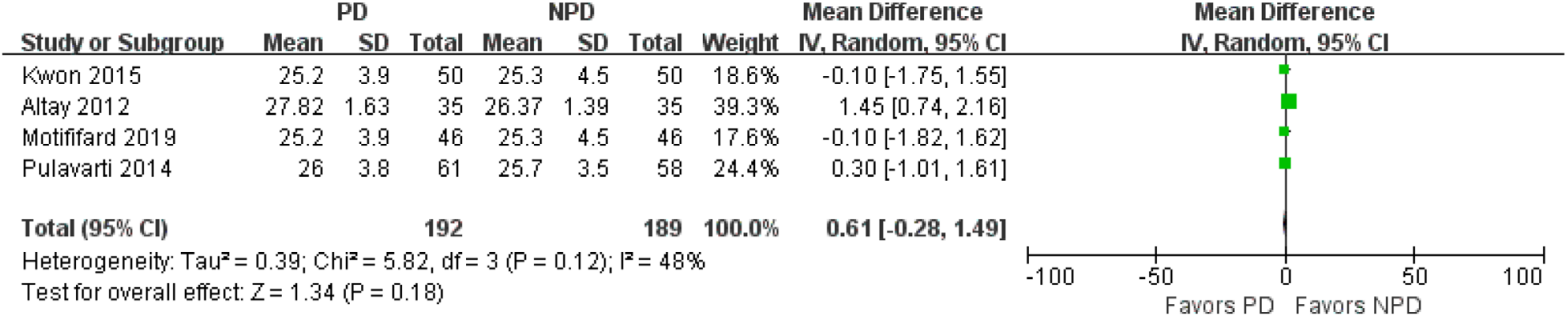
Forest plot of Patellar score (PS).

Four studies involving 381 patients compared the PS between two groups. There was no significant association between PD and increased PS (mean difference, 0.61; 95% CI, −0.28 to 1.49; *P* = 0.18; Fig. 7). Subgroup analysis showed the results remained unchanged in the subgroups of 3‐month, 12‐month and over 12‐month follow‐up (Table 3).

#### *American Knee Society Knee Score (KSS Knee)*

**Fig. 8:**
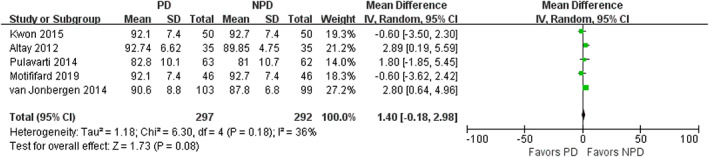
Forest plot of American Knee Society knee score (KSS knee).

Five studies involving 589 patients evaluated the KSS knee, and found no significant difference between two groups (mean difference, 1.40; 95% CI, −0.18 to 2.98; *P* = 0.08; Fig. 8). The results remained unchanged with different follow‐up times. (Table 3).

#### *American Knee Society Function Score (KSS Function)*

**Fig. 9:**
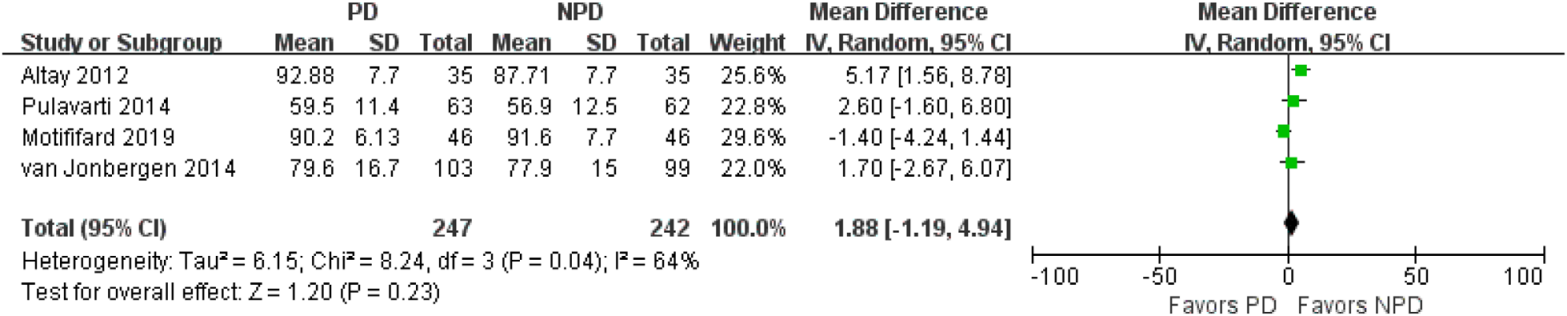
Forest plot of American Knee Society function score (KSS function).

Four studies involving 489 patients evaluated the KSS function, and found no significant difference between the two groups (mean difference, 1.88; 95% CI, −1.19 to 4.94; *P* = 0.23; Fig. 9). In the subgroup analysis, KSS function had an increased mean difference of 4.67 in favor of PD at 3‐month follow‐up (95% CI, 2.07 to 7.26; *P* < 0.001) and increased mean difference of 3.66 in favor of PD after 12‐month follow‐up (95% CI, 0.29 to 7.04; *P* = 0.03). There was no difference at follow‐up time of 12 months (Table 3).

#### *Oxford Knee Score (OKS)*

**Fig. 10:**
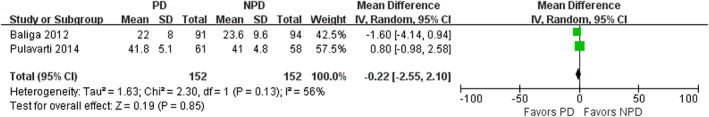
Forest plot of Oxford knee score (OKS).

There was no significant difference in OKS between the two groups (mean difference, −0.22; 95% CI, −2.55 to 2.10; *P* = 0.85; Fig. 10). No significant difference was observed in the subgroup analysis (Table 3).

#### *Postoperative Complication and Reoperation*

**Fig. 11:**
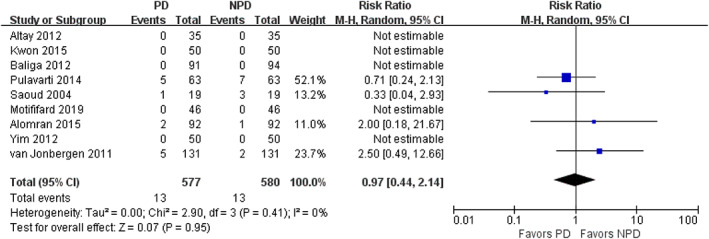
Forest plot of postoperative complication.

**Fig. 12:**
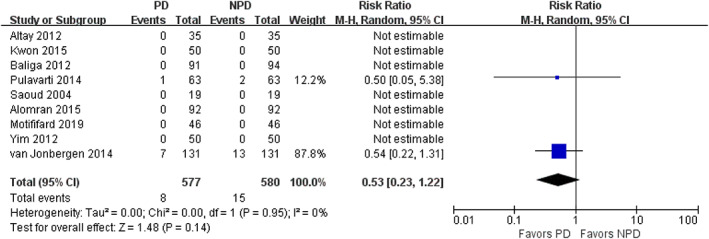
Forest plot of postoperative reoperation.

Nine studies with 1157 patients reported postoperative complication and reoperation. Five studies found no complication occurred in either group and seven studies found no reoperation occurred in either group. There was no significant difference in complication rate (RR = 0.97; 95% CI, 0.44 to 2.14; *P* = 0.95; Fig. 11) and in reoperation rate (RR = 0.53; 95% CI, 0.23 to 1.22; *P* = 0.14; Fig. 12) between the PD group and non‐PD group. No complication related to the patella, such as patellar osteonecrosis, fracture, or dislocation, was reported in the included study.

### *Subgroup Analysis of Depths of Electrocautery*

**Fig. 13:**
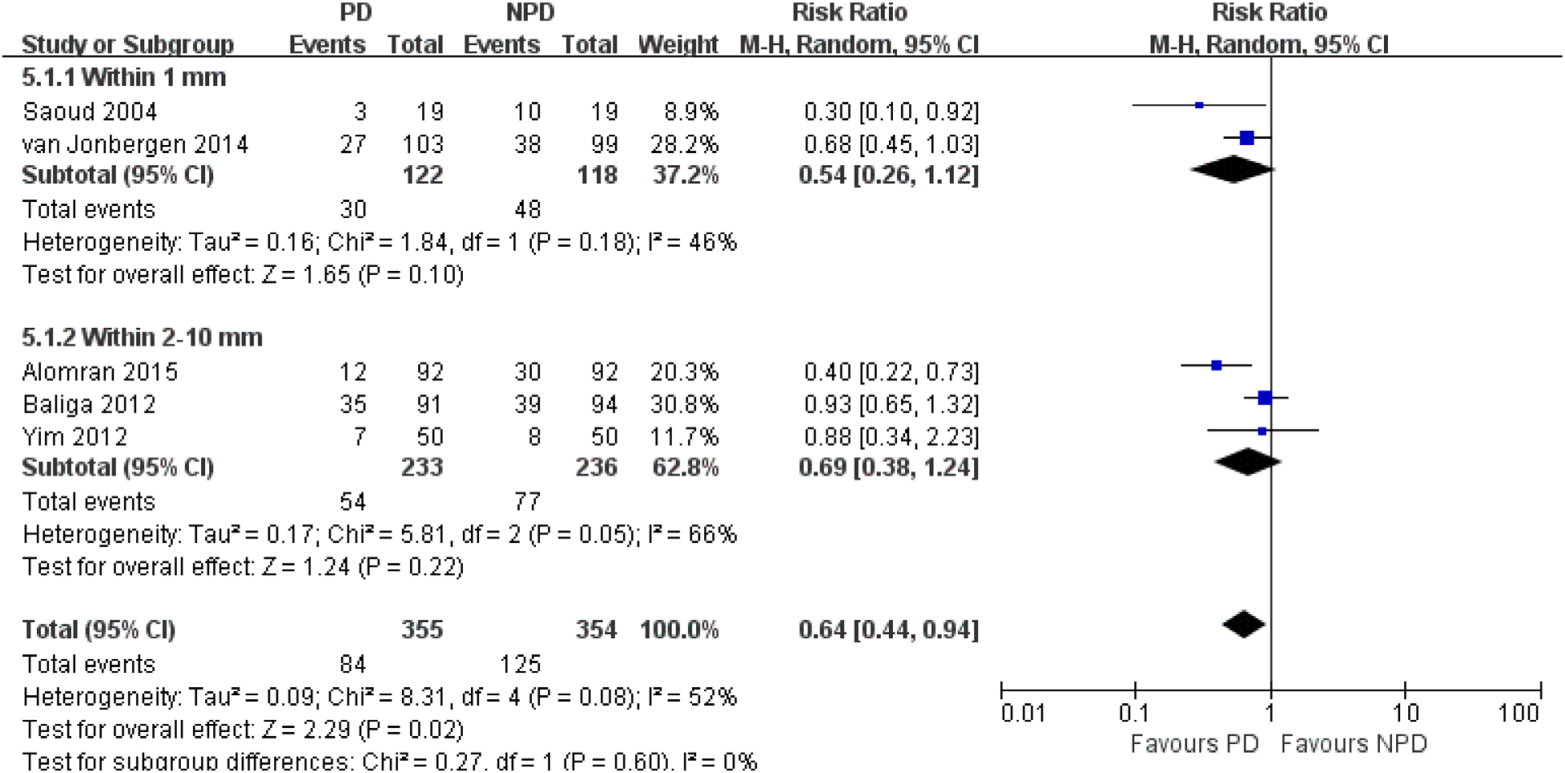
Forest plot of incidence of AKP in subgroup analysis of different depths of electrocautery.

**Fig. 14:**
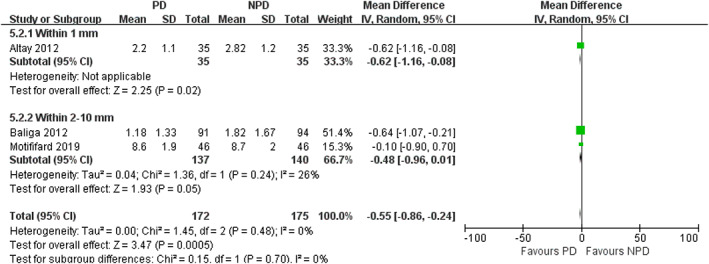
Forest plot of VAS for AKP in subgroup analysis of different depths of electrocautery.

**Fig. 15:**
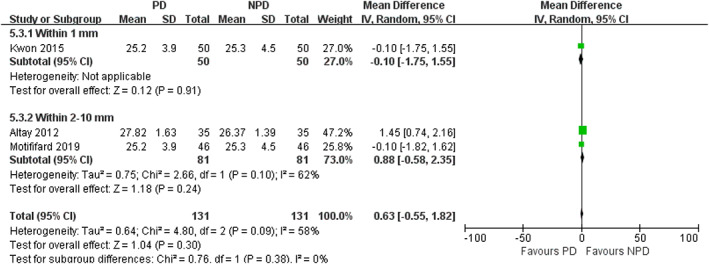
Forest plot of PS in subgroup analysis of different depths of electrocautery.

**Fig. 16:**
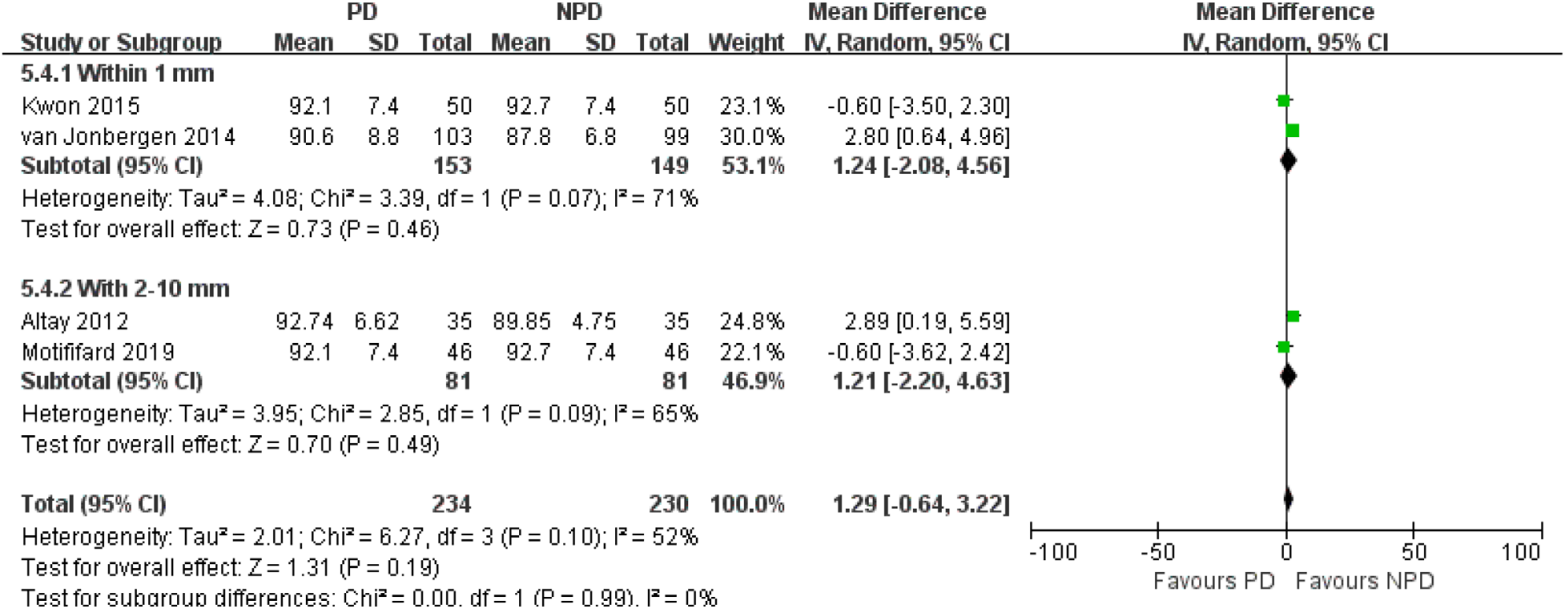
Forest plot of KSS knee in subgroup analysis of different depths of electrocautery.

**Fig. 17:**
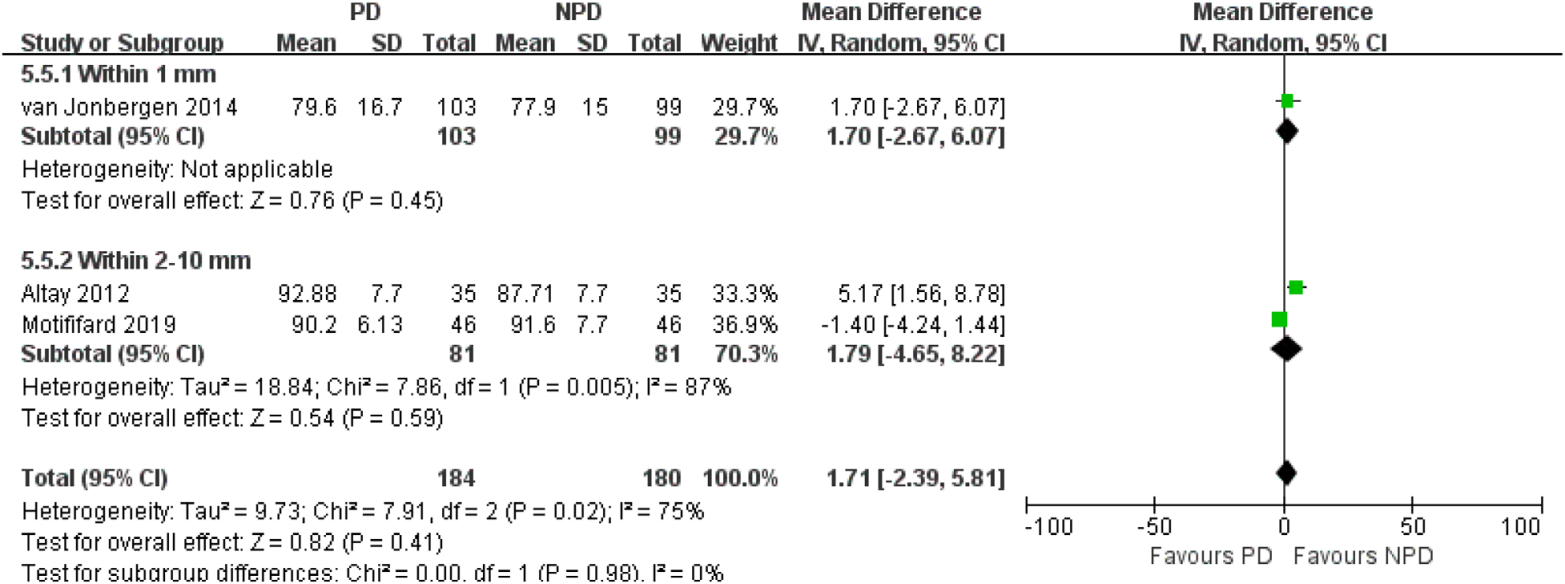
Forest plot of KSS function in subgroup analysis of different depths of electrocautery.

In subgroup analysis of different depths of electrocautery, we found no significant difference in the incidence of AKP between PD and non‐PD patients (within 1 mm subgroup: RR = 0.54; 95% CI, 0.26 to 1.12; *P* = 0.10; within 2–10 mm subgroup: RR = 0.69; 95% CI, 0.38 to 1.24; *P* = 0.22; Fig. 13). In within 1 mm subgroup, PD was associated with a reduction in VAS (mean difference, −0.62; 95% CI, −1.16 to −0.08; *P* = 0.02; Fig. 14). In within 2–10 mm subgroup, no significant difference was observed in VAS between the PD and non‐PD group (mean difference, −0.48; 95% CI, −0.96 to 0.01; *P* = 0.05; Fig. 14). There was no significant association between PD and increased PS in both subgroups (within 1 mm subgroup: mean difference, −0.10; 95% CI, −1.75 to 1.55; *P* = 0.91; within 2–10 mm subgroup: mean difference, 0.88; 95% CI, −0.58 to 2.35; *P* = 0.24; Fig. 15). There was no significant association between PD and increased KSS in both subgroups (within 1 mm subgroup: mean difference, 1.24; 95% CI, −2.08 to 4.56; *P* = 0.46; within 2–10 mm subgroup: mean difference, 1.21; 95% CI, −2.20 to 4.63; *P* = 0.49; Fig. 16). PD was not associated with increased KSS function in both subgroups (within 1 mm subgroup: mean difference, 1.70; 95% CI, −2.67 to 6.07; *P* = 0.45; within 2–10 mm subgroup: mean difference, 1.79; 95% CI, −4.65 to 8.22; *P* = 0.59; Fig. 17).

### *Publication Bias*

**Fig. 18:**
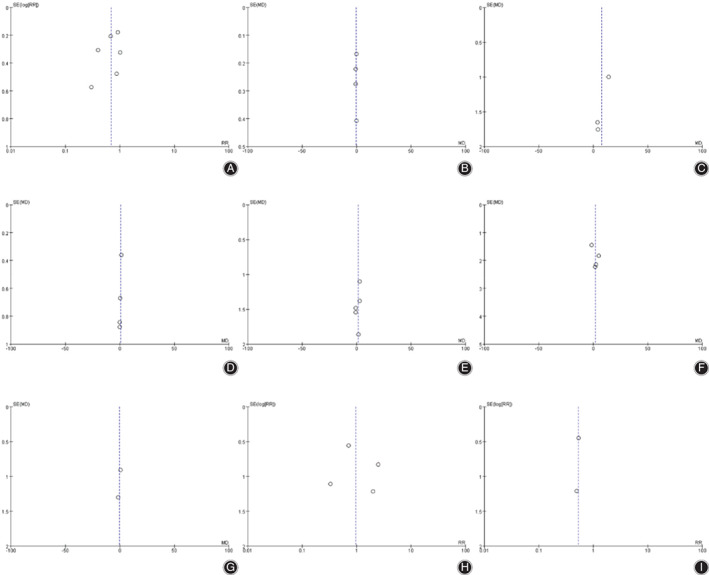
Funnel plots for detecting publication bias. A: Anterior knee pain (AKP). B: Visual analogue scale (VAS). C: Range of motion (ROM). D: Patellar score (PS). E: American Knee Society knee score (KSS knee). F: American Knee Society function score (KSS function). G: Oxford knee score (OKS). H: Postoperative complication. I: Postoperative reoperation.

The funnel plots for the nine outcomes are shown in Figure 18. No significant funnel plot asymmetry was detected, suggesting no evidence of publication bias.

## Discussion

The most important finding of this study was that PD can significantly relieve AKP within 12 months after TKA. Incidence and severity of AKP were both lower in the PD group than in the non‐PD group at 3‐ and 12‐month follow‐up, but the difference was not significant after 12‐month follow‐up. The PD group showed significantly higher ROM than the non‐PD group after 12‐month follow‐up. There was no strong evidence to support the idea that PD had a positive effect on functional scores after TKA. No specific complication directly or indirectly related to PD was found, and the overall complication rate and reoperation rate were comparable between PD and non‐PD group. As a result, the use of PD is recommended in primary TKA without patellar resurfacing to reduce incidence and severity of AKP at the early stage of postoperative period. We believe that the present study, which includes the largest number of RCTs available in the literature, represents the most comprehensive evaluation comparing PD and non‐PD, provides an updated synthesis research of the latest findings, and guides clinical practice in the management of the patella during a TKA procedure reliably.

Total joint arthroplasty is considered to be the successful treatment of choice for end‐stage joint osteoarthritis, which can provide excellent pain relief^26^. The presence of AKP has been a common and persistent complaint after TKA, and can result in dissatisfaction and low quality of life of patients^1^, ^2^. A number of factors were believed to potentially contribute to the occurrence of AKP, including patellofemoral degeneration^27^, patellofemoral joint instability^28^, abnormal patellofemoral joint loading^29^, ^30^, ^31^, and prosthetic design^32^. There is no consensus on the optimal management of the patella during a TKA procedure, and which treatment can be effective to prevent the development of AKP remains unknown. The use of patellar resurfacing and its effect on APK are still controversial. The most recent meta‐analysis involving 20 RCTs failed to show the association between patellar resurfacing and superior clinical outcomes, such as lower incidence of AKP and higher knee function scores^3^. Thus, patellar retention may be routinely recommended to reduce surgical time, decrease hospital cost and avoid severe complications related to patellar resurfacing^33^, ^34^. However, AKP still occurred in 22.6% of the patients without patellar resurfacing^3^. Since substance P nociceptive afferent fibers were found to be rich in the peripatellar soft tissues, PD has been recommended by some surgeons to reduce the likelihood and severity of AKP through desensitization or denervation of pain receptors in anterior knee region.

Several previous meta‐analyses comparing PD and non‐PD in TKA without patellar resurfacing showed discordant results; one of them concluded PD had no clear advantage over non‐PD^35^ while others found PD can provide superior postoperative clinical results^16^, ^36^, ^37^. One meta‐analysis performed by Xie *et al* ^17^. investigated the association between PD effects and follow‐up period, and found that PD can significantly relieve AKP and improve knee function of TKA for up to 12 months of follow‐up but not for over 12 months of follow‐up. Including an additional three RCTs, this study confirms that PD is effective for relieving AKP within 12 months of follow‐up but the effect cannot be maintained more than 12 months after surgery. Besides, the result of this study indicates PD patients have no superior knee function to non‐PD patients at any follow‐up time point, which is different from the result of the previous meta‐analysis^17^.

The findings of this study are in accordance with the results of several published RCTs with good design and adequate follow‐up time^13^, ^14^, ^15^, ^25^. The disappeared effect of PD at longer follow‐up times may be explained by the large amount of potential contributors of AKP, as discussed above. Besides, it is noteworthy that the occurrence of AKP itself is a dynamic process. A gradual decline in the incidence of AKP over time was observed in several studies^38^, ^39^, ^40^, which may explain the weakened preventive effect of PD on AKP after longer follow‐up time. Better pain relief at early postoperative stage may enhance the rapid recovery and functional rehabilitation after TKA, contributing to the better ROM in the PD group. In the subgroup analysis of different depths of electrocautery, there is almost no difference in AKP and knee function between the PD and non‐PD group. The reason for this finding is still unclear. Limited number of studies and participants included in each subgroup may explain the insignificant difference.

Concerns about PD‐related complications still remain. Compromised patellar blood supply caused by circumpatellar electrocautery and concomitant soft tissue injury were theoretically associated with patellar osteonecrosis and fracture^41^, ^42^. However, no specific complication directly or indirectly related to PD, such as patellar osteonecrosis, patellar fracture, patellar clunk, patellar dislocation, or extensor mechanism disruption, has been reported in the included study. No significant difference was observed in the overall complication rates and reoperation rates between the PD and non‐PD group. The majority of the reported complications were superficial wound complications, limited ROM needing manipulation under anesthesia and deep vein thrombosis, which were almost all successfully managed non‐operatively. Van Jonbergen *et al*. reported three cases of secondary patellar resurfacing in the PD group and seven cases in the non‐PD group at a mean follow‐up of 44 months^15^. The indication for secondary patellar resurfacing in the study was severe disabled AKP after 12‐month follow‐up. Besides, some surgeons hypothesized that the proprioception disturbance of the patella caused by PD might induce abnormal patellofemoral joint loading^42^, which was believed to be a risk factor for the occurrence of AKP^31^. However, the result of our study did not support the hypothesis but instead the PD can decrease the incidence and severity of AKP within 1 year after TKA.

This study has several limitations. First, significant heterogeneity was found in the outcomes of PS, KSS function, and OKS, which may be explained by the difference in the way the outcome was measured. In addition, some potential risk factors of AKP, such as race of patients, ratio of female to male, and age group, were inconsistent among included studies, which may lead to significant clinical heterogeneity. Second, not all studies provided follow‐up data for each outcome. Thus, the number of studies and sample size were limited for several outcomes, which made it difficult to perform further subgroup analysis and sensitivity analysis. Third, there is notably unclear risk of bias in several included studies due to poorly described methodology. Considering the limitations in this meta‐analysis, additional high‐quality RCTs with adequate follow‐up time are required to strengthen the evidence.

### *Conclusion*

In conclusion, PD can decrease the incidence and severity of AKP within 12 months after TKA, but the effect cannot be maintained after 12‐month follow‐up. Better pain relief at early postoperative stage may improve ROM in the PD group after 12‐month follow‐up. Without significant associated complication and reoperation, the use of PD is recommended in TKA without patellar resurfacing. Additional high‐quality RCTs are required to strengthen the evidence.

## Disclosure Statement

All authors declared no conflict of interest.

## References

[1] Barrack RL , Bertot AJ , Wolfe MW , Waldman DA , Milicic M , Myers L . Patellar resurfacing in total knee arthroplasty. A prospective, randomized, double‐blind study with five to seven years of follow‐up. J Bone Joint Surg Am, 2001, 83: 1376–1381. 11568201

[2] Breugem SJ , Sierevelt IN , Schafroth MU , Blankevoort L , Schaap GR , van Dijk CN . Less anterior knee pain with a mobile‐bearing prosthesis compared with a fixed‐bearing prosthesis. Clin Orthop Relat Res, 2008, 466: 1959–1965. 18523833 10.1007/s11999-008-0320-6 PMC2584251

[3] Teel AJ , Esposito JG , Lanting BA , Howard JL , Schemitsch EH . Patellar resurfacing in primary Total knee arthroplasty: a meta‐analysis of randomized controlled trials. J Arthroplasty, 2019, 34: 3124–3132. 31427130 10.1016/j.arth.2019.07.019

[4] Duan G , Liu C , Lin W , *et al* Different factors conduct anterior knee pain following primary Total knee arthroplasty: a systematic review and meta‐analysis. J Arthroplasty, 2018, 33: 1962–1971. 29398258 10.1016/j.arth.2017.12.024

[5] Picetti GD III , McGann WA , Welch RB . The patellofemoral joint after total knee arthroplasty without patellar resurfacing. J Bone Joint Surg Am, 1990, 72: 1379–1382. 2229117

[6] Wojtys EM , Beaman DN , Glover RA , Janda D . Innervation of the human knee joint by substance‐P fibers. Arthroscopy, 1990, 6: 254–263. 1702291 10.1016/0749-8063(90)90054-h

[7] Dye SF . The pathophysiology of patellofemoral pain: a tissue homeostasis perspective. Clin Orthop Relat Res, 2005, 436: 100–110. 10.1097/01.blo.0000172303.74414.7d 15995427

[8] van Jonbergen H‐PW , Barnaart AF , Verheyen CC . A Dutch survey on circumpatellar electrocautery in total knee arthroplasty. Open Orthop J, 2010, 4: 201–203. 21228917 10.2174/1874325001004010201 PMC3019582

[9] Baliga S , McNair CJ , Barnett KJ , MacLeod J , Humphry RW , Finlayson D . Does circumpatellar electrocautery improve the outcome after total knee replacement?: a prospective, randomised, blinded controlled trial. J Bone Joint Surg Br, 2012, 94: 1228–1233. 22933495 10.1302/0301-620X.94B9.27662

[10] Kwon SK , Nguku L , Han CD , Koh YG , Kim DW , Park KK . Is electrocautery of patella useful in patella non‐resurfacing total knee arthroplasty?: a prospective randomized controlled study. J Arthroplasty, 2015, 30: 2125–2127. 26100474 10.1016/j.arth.2015.05.057

[11] Altay MA , Erturk C , Altay N , Akmese R , Isikan UE . Patellar denervation in total knee arthroplasty without patellar resurfacing: a prospective, randomized controlled study. Orthop Traumatol Surg Res, 2012, 98: 421–425. 22552314 10.1016/j.otsr.2012.03.002

[12] Alomran A . Effect of patellar denervation on mid‐term results after non‐resurfaced total knee arthroplasty. A randomised, controlled trial. Acta Orthop Belg, 2015, 81: 609–613. 26790781

[13] Pulavarti RS , Raut VV , McLauchlan GJ . Patella denervation in primary total knee arthroplasty ‐ a randomized controlled trial with 2 years of follow‐up. J Arthroplasty, 2014, 29: 977–981. 24291230 10.1016/j.arth.2013.10.017

[14] van Jonbergen HP , Scholtes VA , van Kampen A , Poolman RW . A randomised, controlled trial of circumpatellar electrocautery in total knee replacement without patellar resurfacing. J Bone Joint Surg Br, 2011, 93: 1054–1059. 21768628 10.1302/0301-620X.93B8.26560

[15] van Jonbergen HP , Scholtes VA , Poolman RW . A randomised, controlled trial of circumpatellar electrocautery in total knee replacement without patellar resurfacing: a concise follow‐up at a mean of 3.7 years. Bone Joint J, 2014, 96‐b: 473–478. 10.1302/0301-620X.96B4.32118 24692613

[16] Zhang P , Liu H , Yan WS , Wang WL . Is patellar denervation necessary in total knee arthroplasty without patellar resurfacing? Knee Surg Sports Traumatol Arthrosc, 2016, 24: 2541–2549. 26423054 10.1007/s00167-015-3811-5

[17] Xie X , Pei F , Huang Z , Tan Z , Yang Z , Kang P . Does patellar denervation reduce post‐operative anterior knee pain after total knee arthroplasty? Knee Surg Sports Traumatol Arthrosc, 2015, 23: 1808–1815. 25758982 10.1007/s00167-015-3566-z

[18] Liberati A , Altman DG , Tetzlaff J , *et al* The PRISMA statement for reporting systematic reviews and meta‐analyses of studies that evaluate healthcare interventions: explanation and elaboration. BMJ, 2009, 339: b2700. 19622552 10.1136/bmj.b2700 PMC2714672

[19] Shea BJ , Reeves BC , Wells G , *et al* AMSTAR 2: a critical appraisal tool for systematic reviews that include randomised or non‐randomised studies of healthcare interventions, or both. BMJ, 2017, 358: j4008. 28935701 10.1136/bmj.j4008 PMC5833365

[20] Higgins JP , Altman DG , Gotzsche PC , *et al* The Cochrane collaboration's tool for assessing risk of bias in randomised trials. BMJ, 2011, 343: d5928. 22008217 10.1136/bmj.d5928 PMC3196245

[21] Hozo SP , Djulbegovic B , Hozo I . Estimating the mean and variance from the median, range, and the size of a sample. BMC Med Res Methodol, 2005, 5: 13. 15840177 10.1186/1471-2288-5-13 PMC1097734

[22] Higgins J , Thomas J , Chandler J , *et al* Cochrane Handbook for Systematic Reviews of Interventions Version 6.0 (updated July 2019). London: Cochrane, 2019 Available from: www.training.cochrane.org/handbook.

[23] Saoud AMF . Patellar denervation in non‐patellar resurfacing total knee arthroplasty. Pan Arab J Orthop Trauma, 2004, 8: 25–30.

[24] Yim SJ , Jang MS , Kim WJ , Lee SH , Kang HK . The effect of electrocautery around the patellar rim in patellar non‐resurfacing total knee arthroplasty. Knee Surg Relat Res, 2012, 24: 104–107. 22708111 10.5792/ksrr.2012.24.2.104 PMC3373996

[25] Motififard M , Nazem K , Zarfeshani A , Zarfeshani K . Effect of patellar electrocautery neurectomy on postoperative pain among patients referred for total knee arthroplasty. Adv Biomed Res, 2018, 7: 9. 29456980 10.4103/abr.abr_154_16 PMC5812092

[26] Ding ZC , Xu B , Liang ZM , Wang HY , Luo ZY , Zhou ZK . Limited influence of comorbidities on length of stay after total hip arthroplasty: experience of enhanced recovery after surgery. Orthop Surg, 2020, 12: 153–161. 31885219 10.1111/os.12600 PMC7031546

[27] Rodriguez‐Merchan EC , Gomez‐Cardero P . The outerbridge classification predicts the need for patellar resurfacing in TKA. Clin Orthop Relat Res, 2010, 468: 1254–1257. 19844770 10.1007/s11999-009-1123-0 PMC2853678

[28] Malo M , Vince KG . The unstable patella after total knee arthroplasty: etiology, prevention, and management. J Am Acad Orthop Surg, 2003, 11: 364–371. 14565758 10.5435/00124635-200309000-00009

[29] Smith AJ , Lloyd DG , Wood DJ . Pre‐surgery knee joint loading patterns during walking predict the presence and severity of anterior knee pain after total knee arthroplasty. J Orthop Res, 2004, 22: 260–266. 15013083 10.1016/S0736-0266(03)00184-0

[30] Barrack RL , Schrader T , Bertot AJ , Wolfe MW , Myers L . Component rotation and anterior knee pain after total knee arthroplasty. Clin Orthop Relat Res, 2001, 392: 46–55. 10.1097/00003086-200111000-00006 11716424

[31] van Jonbergen HP , Reuver JM , Mutsaerts EL , Poolman RW . Determinants of anterior knee pain following total knee replacement: a systematic review. Knee Surg Sports Traumatol Arthrosc, 2014, 22: 478–499. 23160846 10.1007/s00167-012-2294-x

[32] Becher C , Heyse TJ , Kron N , *et al* Posterior stabilized TKA reduce patellofemoral contact pressure compared with cruciate retaining TKA in vitro. Knee Surg Sports Traumatol Arthrosc, 2009, 17: 1159–1165. 19305974 10.1007/s00167-009-0768-2

[33] Schiavone Panni A , Cerciello S , Del Regno C , Felici A , Vasso M . Patellar resurfacing complications in total knee arthroplasty. Int Orthop, 2014, 38: 313–317. 24363045 10.1007/s00264-013-2244-3 PMC3923924

[34] Grassi A , Compagnoni R , Ferrua P , *et al* Patellar resurfacing versus patellar retention in primary total knee arthroplasty: a systematic review of overlapping meta‐analyses. Knee Surg Sports Traumatol Arthrosc, 2018, 26: 3206–3218. 29335747 10.1007/s00167-018-4831-8

[35] Cheng T , Zhu C , Guo Y , Shi S , Chen D , Zhang X . Patellar denervation with electrocautery in total knee arthroplasty without patellar resurfacing: a meta‐analysis. Knee Surg Sports Traumatol Arthrosc, 2014, 22: 2648–2654. 23743580 10.1007/s00167-013-2533-9

[36] Li T , Zhou L , Zhuang Q , Weng X , Bian Y . Patellar denervation in total knee arthroplasty without patellar resurfacing and postoperative anterior knee pain: a meta‐analysis of randomized controlled trials. J Arthroplasty, 2014, 29: 2309–2313. 24582160 10.1016/j.arth.2014.01.024

[37] Fan L , Ge Z , Zhang C , *et al* Circumferential electrocautery of the patella in primary total knee replacement without patellar replacement: a meta‐analysis and systematic review. Sci Rep, 2015, 5: 9393. 25801456 10.1038/srep09393 PMC4371101

[38] Arbuthnot JE , McNicholas MJ , McGurty DW , Rowley DI . Total knee replacement and patellofemoral pain. Surgeon, 2004, 2: 230–233. 15570832 10.1016/s1479-666x(04)80006-4

[39] Mahoney OM , McClung CD , dela Rosa MA , Schmalzried TP . The effect of total knee arthroplasty design on extensor mechanism function. J Arthroplasty, 2002, 17: 416–421. 12066269 10.1054/arth.2002.32168

[40] Meftah M , Ranawat AS , Ranawat CS . The natural history of anterior knee pain in 2 posterior‐stabilized, modular total knee arthroplasty designs. J Arthroplasty, 2011, 26: 1145–1148. 21277160 10.1016/j.arth.2010.12.013

[41] Putman S , Boureau F , Girard J , Migaud H , Pasquier G . Patellar complications after total knee arthroplasty. Orthop Traumatol Surg Res, 2019, 105: S43–S51. 29990602 10.1016/j.otsr.2018.04.028

[42] Gupta S , Augustine A , Horey L , Meek RM , Hullin MG , Mohammed A . Electrocautery of the patellar rim in primary total knee replacement: beneficial or unnecessary? J Bone Joint Surg Br, 2010, 92: 1259–1261. 20798444 10.1302/0301-620X.92B9.24467

